# Supplementary material for: iPSC-derived PSEN2 (N141I) astrocytes and microglia exhibit a primed inflammatory phenotype
Source: J Neuroinflammation. 2024 Jan 4;21:7. doi: 10.1186/s12974-023-02951-2 (PMC10765839; doi:10.1186/s12974-023-02951-2)
Supplement: Supplementary file 1 — Additional file 1: Supplementary Methods. Table S1. Primary antibodies used for immunofluorescence. Table S2. Secondary antibodies used for immunofluorescence. Fig. S1. Sanger sequencing chromatograms showing APOE genotyping of codon 112 (rs429358) and codon 158 (rs7412) for all iPSC lines. Yellow highlight indicates the position of the single nucleotide polymorphism. Fig. S2. Immunofluorescence images of iPSCs from three healthy control lines (Ctrl-06, Ctrl-71, Ctrl-88) and three familial AD lines harbouring a PSEN2 (N141I) mutation (fAD-08, fAD-948, fAD-950). The cells were stained for pluripotency markers Nanog (red), Oct 3 (green) and all nuclei were counterstained with DAPI (blue). Scale bars = 50 μm. Fig. S3. Immunofluorescence images of iPSC-derived NPCs from three healthy control lines (Ctrl-06, Ctrl-71, Ctrl-88) and three familial AD lines harbouring a PSEN2 (N141I) mutation (fAD-08, fAD-948, fAD-950). The cells were stained for A the neural progenitor markers Pax-6 (red) and Nestin (green), B a pluripotency marker Oct3 (green) and all nuclei were counterstained with DAPI (blue). Scale bars = 50 μm. Fig. S4. Immunofluorescence images of iPSC-derived astrocytes from three healthy control lines (Ctrl-06, Ctrl-71, Ctrl-88) and three familial AD lines harbouring a PSEN2 (N141I) mutation (fAD-08, fAD-948, fAD-950). The cells were stained for A astrocyte markers GFAP (red) and S100β (green), B the NPC marker nestin (green). All nuclei were counterstained with DAPI (blue). Scale bars = 50 μm. Fig. S5. Transcriptomic analysis of iPSC-derived cell types and primary human fetal astrocytes. A Principal component analysis and B cluster analysis of iPSC-derived astrocytes (black) from healthy control lines (lines 06, 71 & 88) generated in our study and commercially-available primary astrocytes grown in our lab (green) combined with a datasets from Tcw et al (23), including primary astrocytes (purple), iPSC-derived NPCs (light blue), astrocytes (dark blue) and neurons (y [file 12974_2023_2951_MOESM1_ESM.docx]

iPSC-Derived PSEN2 (N141I) Astrocytes and Microglia Exhibit a Primed Inflammatory Phenotype

Michael A. Sullivan^1^, Samuel D. Lane^1^, André D.J. McKenzie^1^, Sarah R. Ball^1^, Margaret Sunde^1^, G. Gregory. Neely^2^, Cesar L. Moreno^2^, Alexandra Maximova^1^, Eryn L. Werry^1, 3, 4*^, Michael Kassiou^3*^

^1^School of Medical Sciences, The Faculty of Medicine and Health, The University of Sydney. ^2^School of Life and Environmental Sciences, Faculty of Science, The University of Sydney. ^3^School of Chemistry, The Faculty of Science, The University of Sydney. ^4^Central Clinical School, Faculty of Medicine and Health, The University of Sydney.

*Co-corresponding author: Michael Kassiou

**Email:** [michael.kassiou@sydney.edu.au](mailto:michael.kassiou@sydney.edu.au)

*Co-corresponding author: Eryn Werry

**Email:** [eryn.werry@sydney.edu.au](mailto:eryn.werry@sydney.edu.au)

**Additional file 1**

**Supplementary Methods**

**Extraction of RNA**

Human control iPSC-derived astrocytes and human fetal astrocytes (Lonza:CC-2565) were plated at 12x10^4^ cells/well of a 6-well plate. Human control iPSC-derived neural precursor cells (NPCs) were plated at 48x10^4^ cells/well of a 6-well plate. Cells were then incubated from 24-48 h. RNA extraction was performed using RNeasy kit (Qiagen:74104). In accordance with the manual, cells were washed with warm sterile PBS and lysed with RLT buffer. The lysate was then homogenized using a QIAshredder spin column. The homogenized lysate underwent DNase I digestion. RNA quality was quantified by utilization of RNA 6000 Nano kit (Agilent) and analysed on a 2100 Bioanalyzer Instrument (Agilent).

**Analysis of RNASeq data**

RNA-seq data was obtained by the Australian Genome Research Facility via the Illumina NovaSeq X system to obtain paired-end read data of iPSC-derived NPCs & astrocytes and human fetal astrocytes (accession: GSE243177). Analysis of the RNAseq data collected was in part analysed following the methods outlined by TCW et al (2017). Read data was aligned to the Ensembl GRCH37.70 human genome using Star Aligner (version 2.7.10a). Gene expression was then quantified as counts using FeatureCounts (Liao et al., 2014, version 2.02). Using the trimmed mean of M-values normalization method (Robinson et al., 2010) read counts were normalized as counts per million in R programming. Via linear regression, data sets were adjusted for sex differences. Differential gene expression between cell types was analysed via linear regression using the limma package (Ritchie et al., 2015). A meta-analysis of primary astrocytes and iPSC-derived neurons (TCW et al., 2017) was conducted by combining raw counts analysed in the same pipeline as described above (accession: GSE97904). Count data was then merged with our data and alone and performed in a similar pipeline as above using R programming.

**Results**

**Table S1** Primary antibodies used for immunofluorescence

| **Target** | **Antibody Species** | **Vendor** | **Product Number** | **Dilution** |
| --- | --- | --- | --- | --- |
| Nestin | mouse | Stem Cell | 60091 | 1:2000 |
| Pax6 | rabbit | Abcam | ab5790 | 1:50 |
| Oct3 | mouse | Stem Cell | 60093.1 | 1:1000 |
| GFAP | rabbit | Abcam | ab7260 | 1:500 |
| S100β | mouse | Sigma Aldrich | S2532 | 1:1000 |
| Iba1 | rabbit | Wako | 019-19741 | 1:500 |
| TREM2 | goat | R&D Systems | AF1828 | 1:100 |
| CX3CR1 | rabbit | Biorad | AHP1589 | 1:250 |

**Table S2** Secondary antibodies used for immunofluorescence

| **Species Reactivity** | **Host** | **Conjugate** | **Vendor** | **Product Number** | **Dilution** |
| --- | --- | --- | --- | --- | --- |
| Mouse | Donkey | Alexa Fluor 488 | ThermoFisher | A-21202 | 1:200 |
| Rabbit | Donkey | Alexa Fluor 594 | ThermoFisher | A-21207 | 1:200 |
| Goat | Donkey | Alexa Fluor 488 | ThermoFisher | A-11055 | 1:200 |


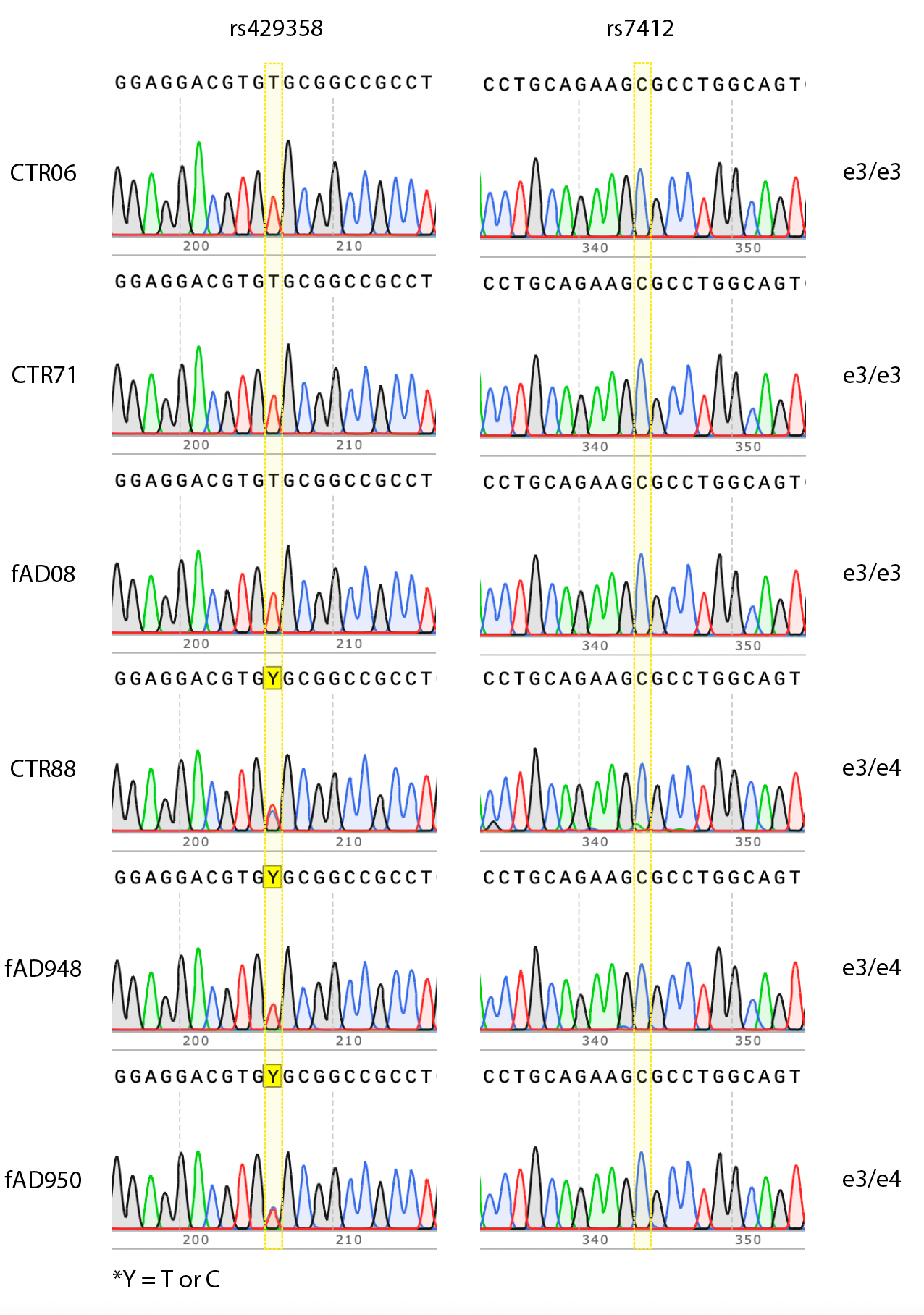


**Fig. S1** Sanger sequencing chromatograms showing APOE genotyping of codon 112 (rs429358) and codon 158 (rs7412) for all iPSC lines. Yellow highlight indicates the position of the single nucleotide polymorphism.

**Fig. S2** Immunofluorescence images of iPSCs from three healthy control lines (Ctrl-06, Ctrl-71, Ctrl-88) and three familial AD lines harbouring a PSEN2 (N141I) mutation (fAD-08, fAD-948, fAD-950). The cells were stained for pluripotency markers Nanog (red), Oct 3 (green) and all nuclei were counterstained with DAPI (blue). Scale bars = 50 μm.


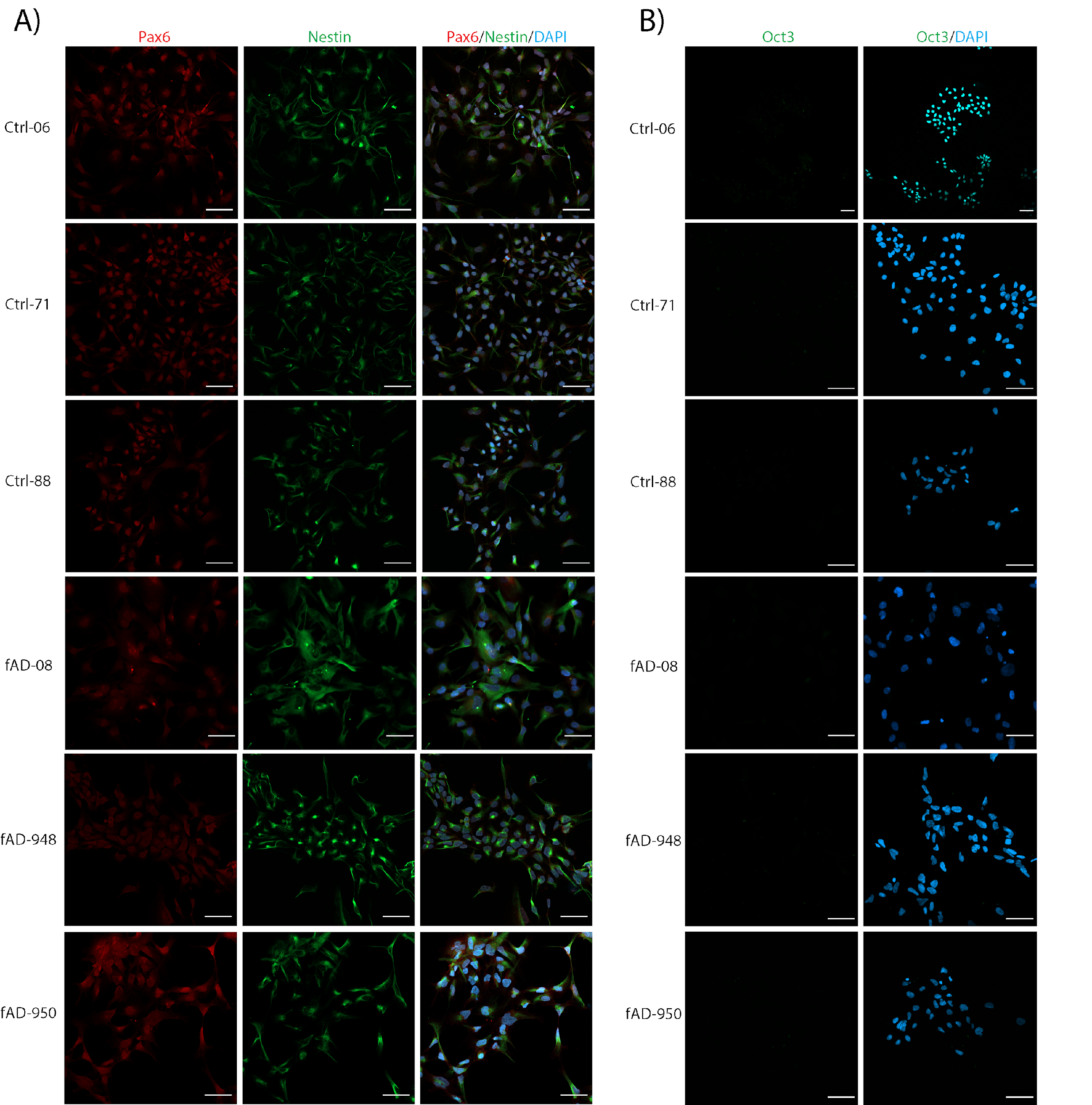


**Fig. S3** Immunofluorescence images of iPSC-derived NPCs from three healthy control lines (Ctrl-06, Ctrl-71, Ctrl-88) and three familial AD lines harbouring a PSEN2 (N141I) mutation (fAD-08, fAD-948, fAD-950). The cells were stained for A) the neural progenitor markers Pax-6 (red) and Nestin (green), B) a pluripotency marker Oct3 (green) and all nuclei were counterstained with DAPI (blue). Scale bars = 50 μm.


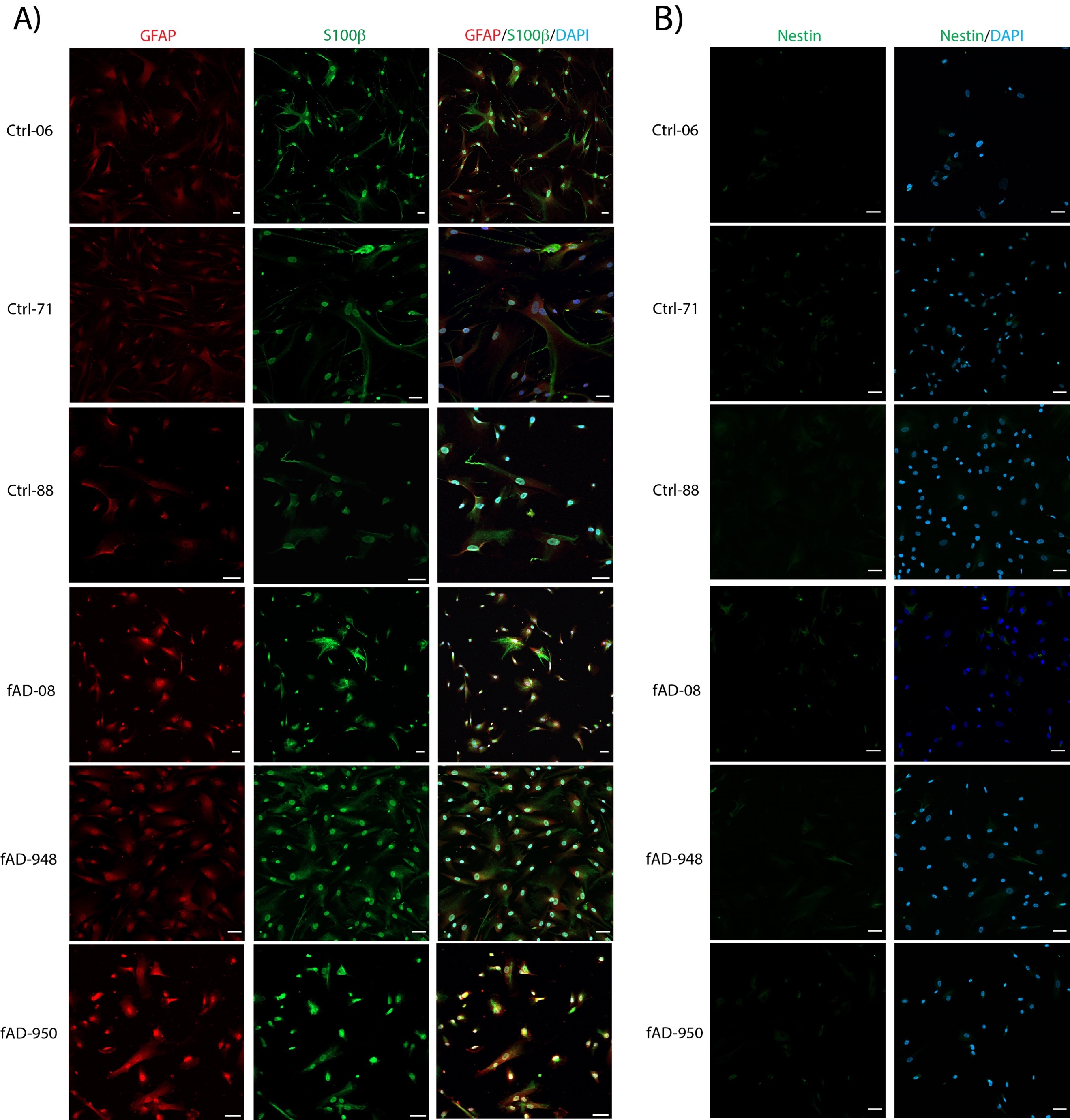


**Fig. S4** Immunofluorescence images of iPSC-derived astrocytes from three healthy control lines (Ctrl-06, Ctrl-71, Ctrl-88) and three familial AD lines harbouring a PSEN2 (N141I) mutation (fAD-08, fAD-948, fAD-950). The cells were stained for A) astrocyte markers GFAP (red) and S100β (green), B) the NPC marker nestin (green). All nuclei were counterstained with DAPI (blue). Scale bars = 50 μm.


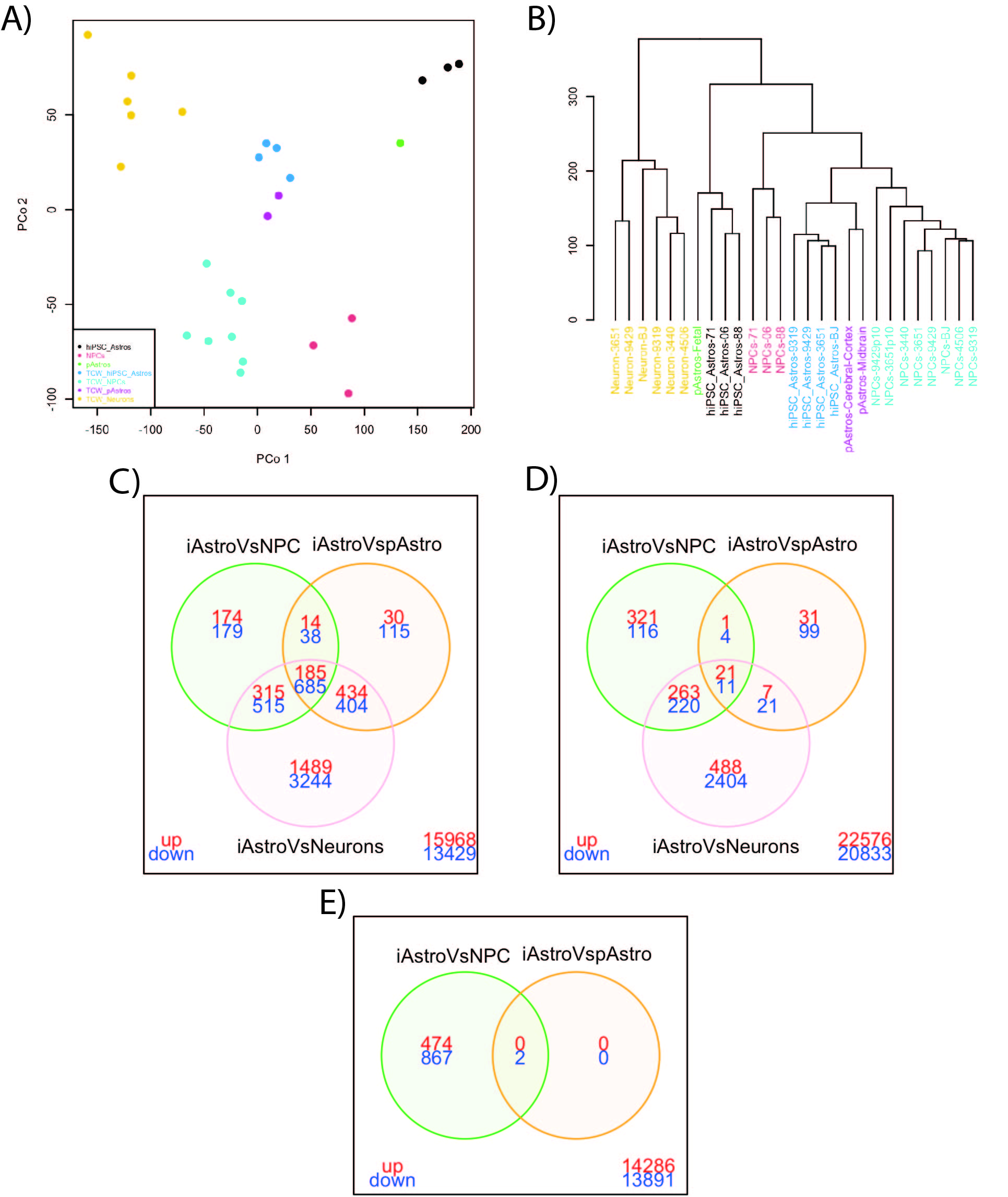


**Fig. S5** Transcriptomic analysis of iPSC-derived cell types and primary human fetal astrocytes. A) Principal component analysis and B) cluster analysis of iPSC-derived astrocytes (black) from healthy control lines (lines 06, 71 & 88) generated in our study and commercially-available primary astrocytes grown in our lab (green) combined with a datasets from Tcw et al (23), including primary astrocytes (purple), iPSC-derived NPCs (light blue), astrocytes (dark blue) and neurons (yellow). Contrast matrix of differential gene expression between cell types comparing datasets C) our iPSC-derived Astrocytes vs. Tcw et al’s primary astrocyte and iPSC-derived neurons datasets, D) Tcw et al’s dataset alone, E) our data alone.


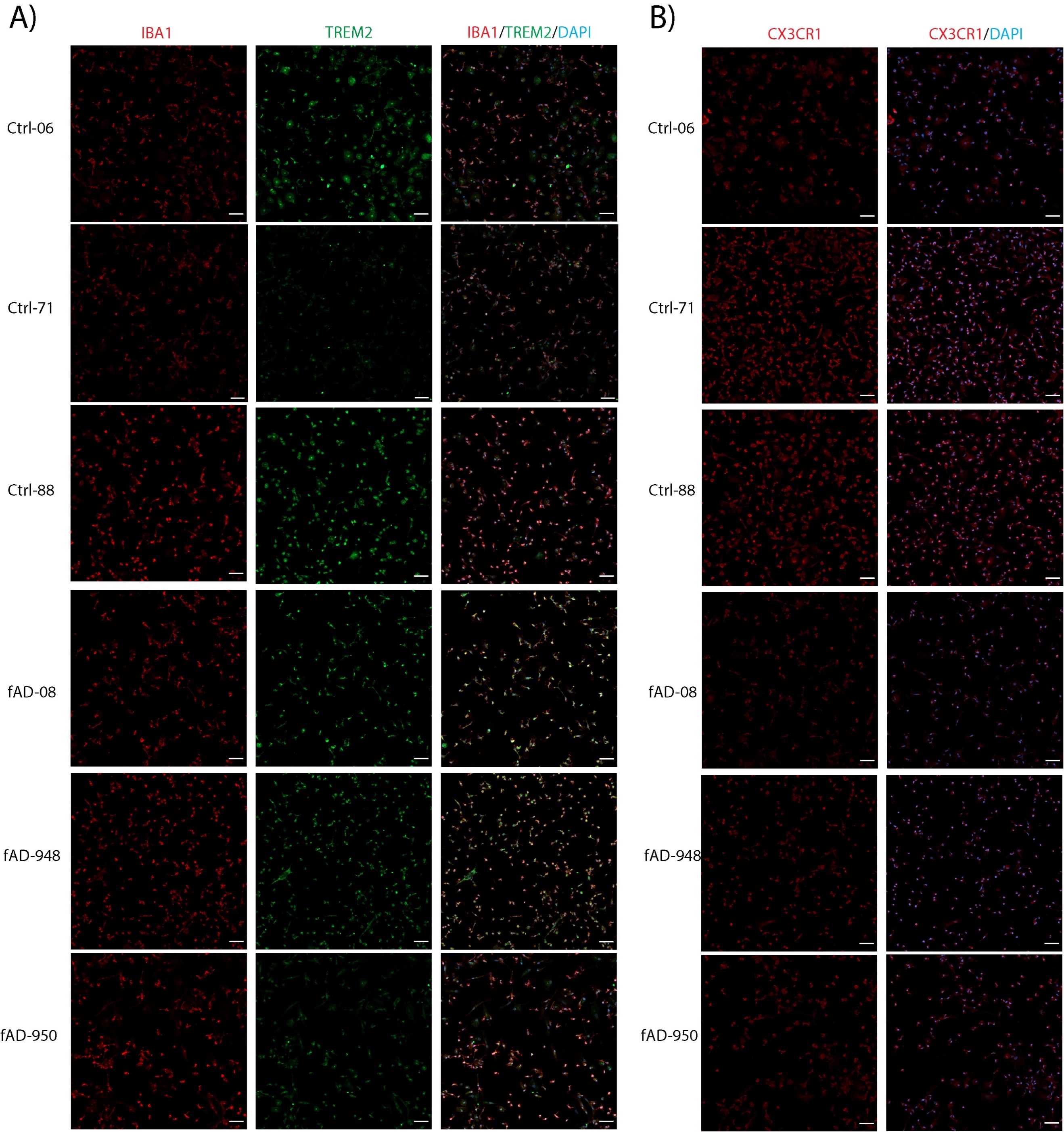


**Fig. S6** Immunofluorescence images of iPSC-derived microglia-like cells from three healthy control lines (Ctrl-06, Ctrl-71, Ctrl-88) and three familial AD lines harbouring a PSEN2 (N141I) mutation (fAD-08, fAD-948, fAD-950). Images show cells stained for A) the microglial markers IBA1 (red), TREM2 (green), B) CX3CR1 (red) and all nuclei were counterstained with DAPI (blue). Scale bars = 50 μm.


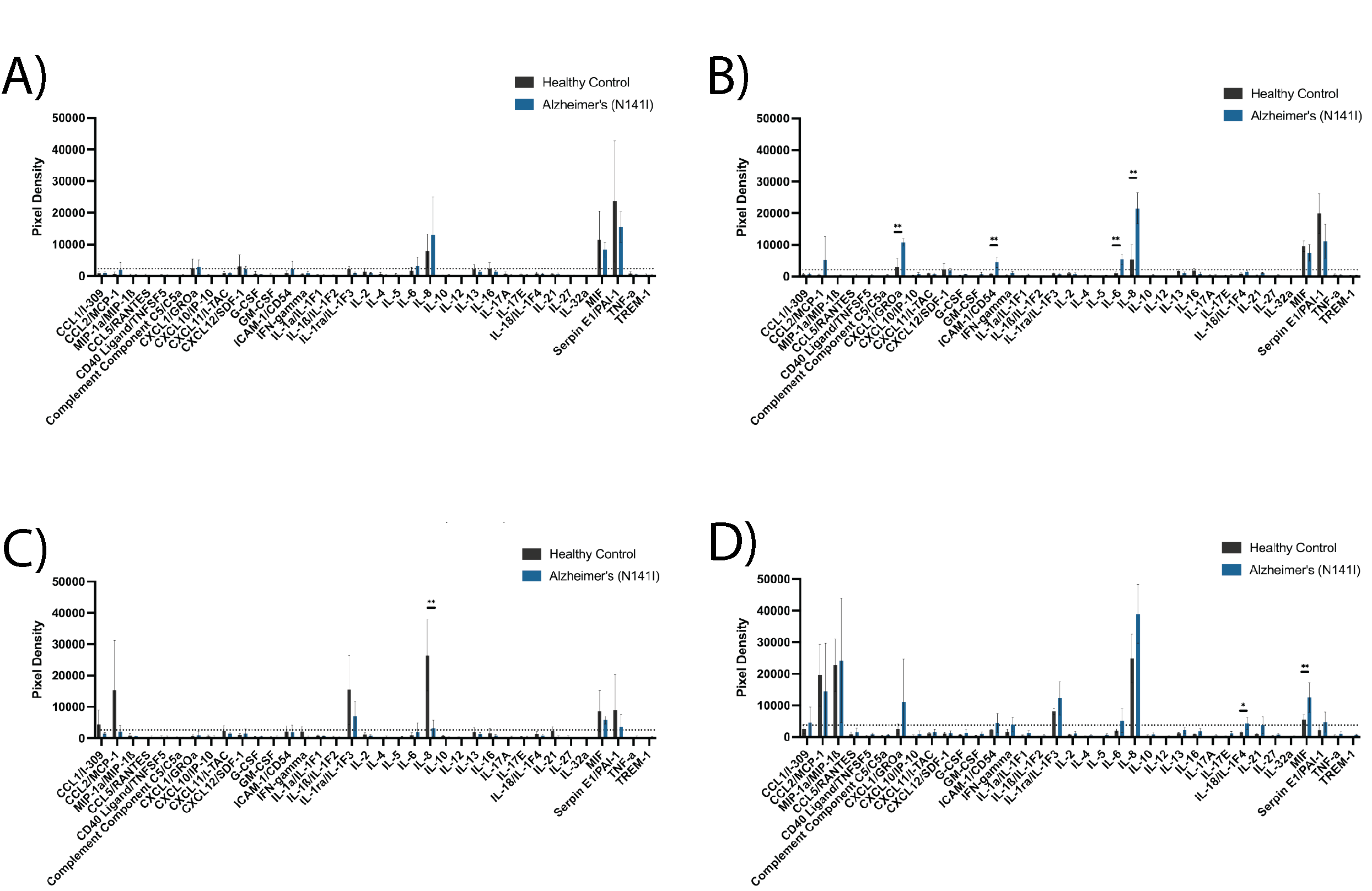


**Fig. S7** Multi-cytokine array of Alzheimer’s or healthy iPSC-derived astrocytes A) basally and B) after 24 h exposure to 10 μM Aβ_42_ and iPSC-derived microglia-like cells C) basally and D) after 24 h exposure to 10 μM Aβ_42_. The figure displays the mean ± SD of three cell lines with the average of two experimental duplicates per line. Multiple unpaired, non-parametric Mann-Whitney t-tests adjusting for a 0.05 false discovery rate were used to test whether there were statistically significant differences between mean cytokine/chemokine release of AD-derived and healthy control astrocytes and microglia-like cells (* p < 0.05, ** p < 0.01). Cytokines that yielded an average intensity value less than 10% of the maximum (represented by the dotted line) were considered background and not included in the statistical analysis.


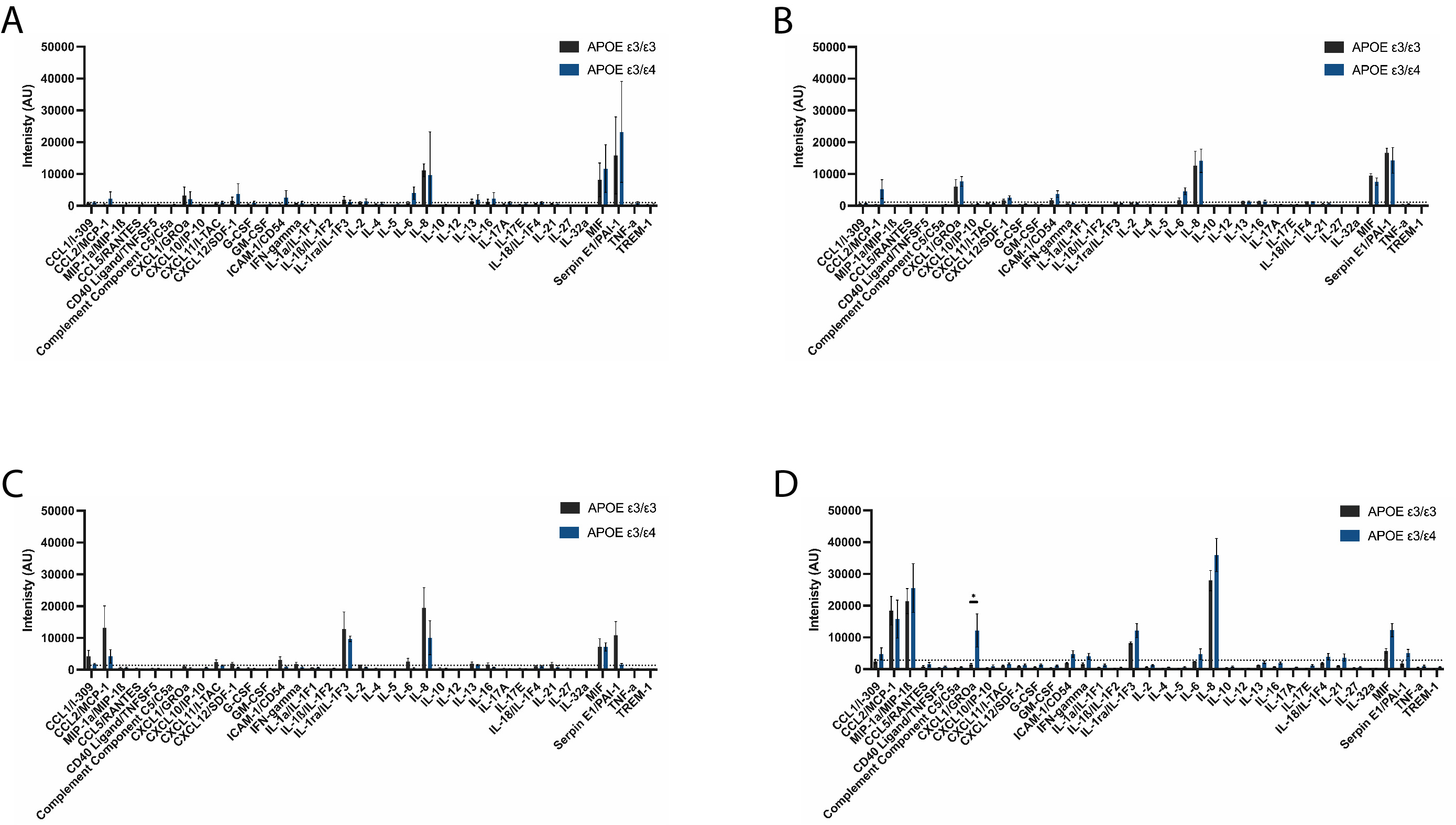


**Fig. S8** Multi-cytokine array of APOE ε3/ε3 and APOE ε3/ε4 iPSC-derived astrocytes A) basally and B) after 24 h exposure to 10 μM Aβ_42_ and iPSC-derived microglia-like cells C) basally and D) after 24 h exposure to 10 μM Aβ_42_. The figure displays the mean ± SD of three cell lines with the average of two experimental duplicates per line. Multiple unpaired, non-parametric Mann-Whitney t-tests adjusting for a 0.05 false discovery rate were used to test whether there were statistically significant differences between mean cytokine/chemokine release of APOE ε3/ε3 and APOE ε3/ε4 astrocytes and microglia-like cells (* p < 0.05). Cytokines that yielded an average intensity value less than 10% of the maximum (represented by the dotted line) were considered background and not included in the statistical analysis.


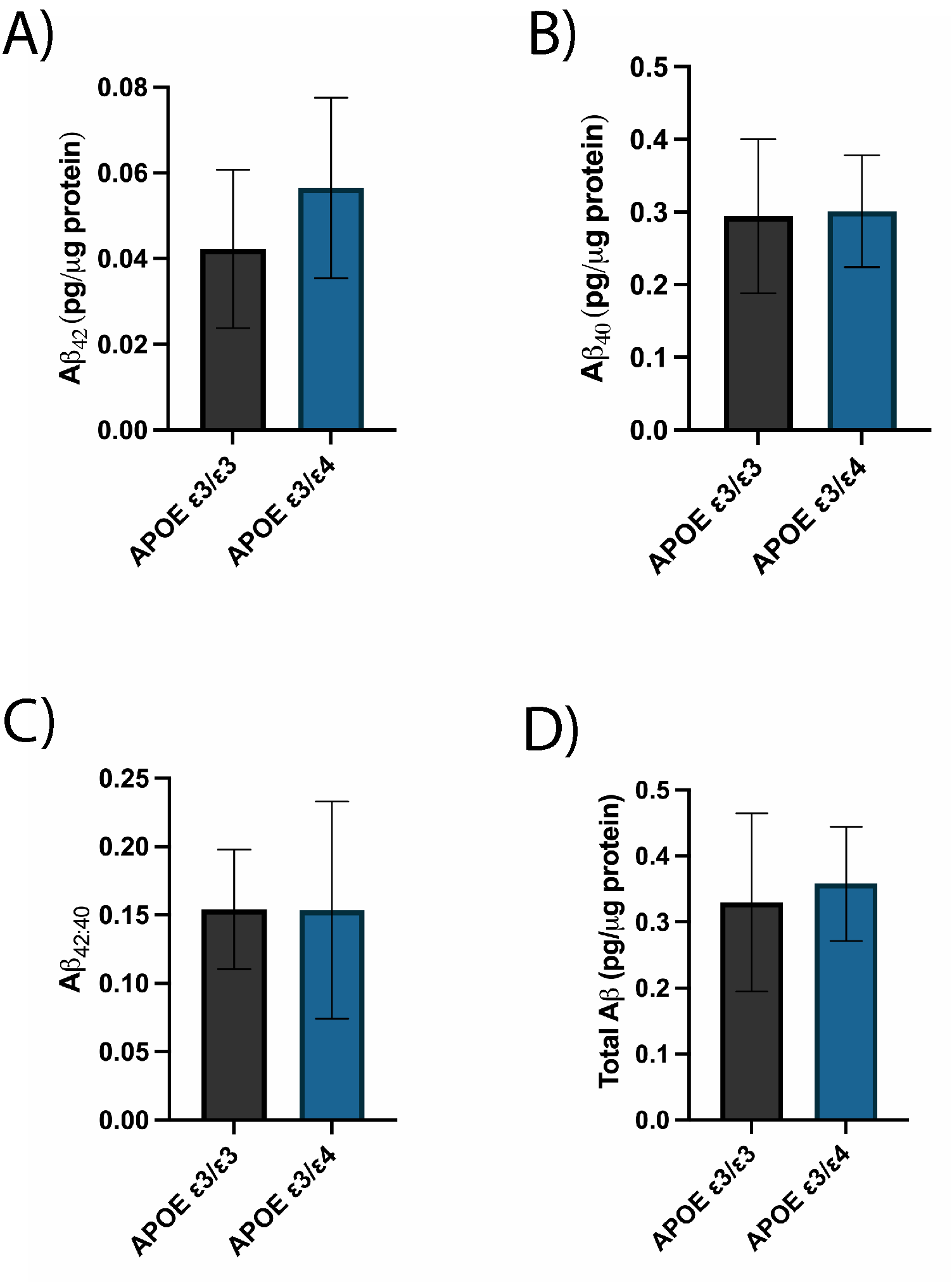


**Fig. S9** Concentrations of A) Aβ_42_, B) Aβ_40_, C) the ratio of Aβ_42:40_ and D) total Aβ protein quantified from iPSC-derived astrocyte supernatants 72 h after plating. Secreted Aβ concentrations were measured using a highly-sensitive ELISA and normalised to total protein concentration determined by BCA. The figure displays the mean ± SD of three cell lines with n ≥ 2 independent experiments per line. A post-hoc unpaired t-test was used to test whether there were statistically significant differences between mean of APOE ε3/ε3 and APOE ε3/ε4 astrocytes.
